# Effects of alcohol on the morphological and structural changes in oral mucosa

**DOI:** 10.12669/pjms.294.3696

**Published:** 2013

**Authors:** Lin Feng, Lili Wang

**Affiliations:** 1 Lin Feng, Oral Medical Research Center, Chinese PLA General Hospital, Beijing 100853, P. R. China.; 2 Lili Wang, Department of Prosthodontics, Affiliated Stomatological Hospital of LMU, Jinzhou 121000, P. R. China.

**Keywords:** Alcoholism, Morphology, Oral mucosa

## Abstract

***Objective:*** To investigate the morphological and structural changes of oral mucosa under the influence of alcohol.

***Methods: ***Sixty male and female specimens (42 males and 18 females) who died of chronic alcoholism were selected in this study. The specimens (5-7 mm) were sliced by the morphological-histological detection method, and stained by the HE and Spielmeyer (myelin staining) protocols respectively. Then five immune peroxidase chemical reaction tests were performed.

***Results: ***10% of the tissue sections had epithelial hyperplasia points with hyperkeratosis and acanthosis. 90% of the sections had epithelial atrophy points with different degrees of damage, and had moderate infiltration of lymphocytes-macrophages in the basal oral mucosa simultaneously. For the tissue sections of patients who died of cardiovascular diseases with a history of alcoholism, about a half showed that extensive necrotic points were observed in different parts of oral mucosa, accompanied by a secondary infection. Approximately 15% of the sections had more dense and homogeneous necrotic tissues with microbial colonization, and the necrotic focus of 5% of the sections was located above the epithelial tissue, which was not distinctively different from other tissues. 48% of the sections were subjected to small nerve bundles with jeopardized deep oral mucosa, accompanied by necrosis of neuron axon and its myelin membrane.

***Conclusion: ***The findings of this study show that drinking alcohol over an extended time may lead to carcinogenic changes in oral mucosa.

## INTRODUCTION

From the viewpoint of epidemiology, chronic alcoholism is an important factor in the development of oral and its mucosal diseases. Many studies have shown that ethanol also plays a key role in the progress of cancerous lesions of oral mucosa. However, the specific pathological process remains partially unclear, which may be attributed to that ethanol *per se* is not a "carcinogen", but ethanol can increase the "permeability" of oral mucosa, resulting in epithelial tissue atrophy. Besides, alcohol is able to decompose the lipid composition of the outer epithelial membrane of mucosal tissue, which augments the "susceptibility" of oral mucosa to other carcinogens. In addition, ethanol can also act on the large and small salivary secreting glands in oral cavity to increase saliva secretion and viscosity. The detailed function of alcohol is still undefined hitherto. Meanwhile, the dangers of drinking alcohol to human body as well as the influence on oral mucosa remain unknown.^1^^,^^2^

## METHODS

**Fig.1:**
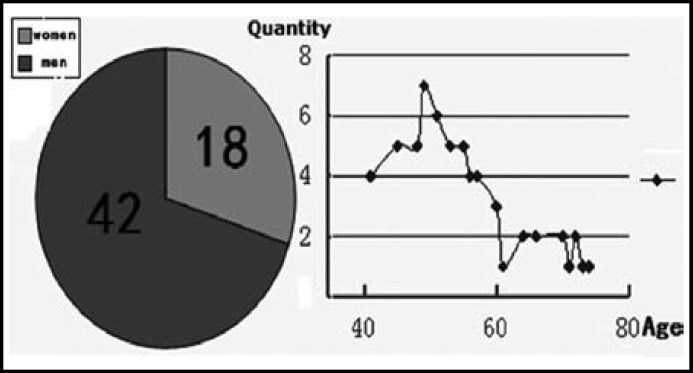
Genders and ages of the corpses from whom specimens were taken

Sixty male and female specimens (42 males and 18 females) who mainly died of chronic alcoholism and alcoholic cirrhosis were selected in this study, of which the ages ranged between 41 and 74 years old (Fig.1). The sample selection and research were completed with the joint efforts of the forensic clinical laboratory of the Military Medical Academy in St. Petersburg and this Academy under the agreement of "Scientific Research". This study was performed in accordance with relevant laws.

The tissue with the size of 1×3 cm was taken from the oral mucosa as the specimen. The position was selected in the junction region between the attached mucosa of upper and lower alveolus and buccal mucosa and lip mucosa. All specimens were immersed in 10% formalin solution, and then sent to the Pathological Testing Laboratory of St. Petersburg Medical University, during which all specimens were not polluted and undermined. After being labeled, all specimens were being added with paraffin and prepared into 5-7 mm thick specimen slices, then stained with HE (artificial hematoxylin and eosin), and the Van-Gieson method (VG), Mallory method (phosphotungstic acid hematoxylin) and Spielmeyer method (myelin staining) were used respectively. Then various immune peroxidase chemical reaction tests were performed to determine the presence of leukocytes. The reactions include: (1) reaction with common leukocyte antigen (LCA); (2) reaction between T-lymphocyte and B-lymphocyte (CD-3, CD-20); (3) reaction with T-helper cell (CD-4); (4) reaction with T-suppressing cell (CD-8); (5) reaction with T-natural killer cell (CD-56).

**Fig.2:**
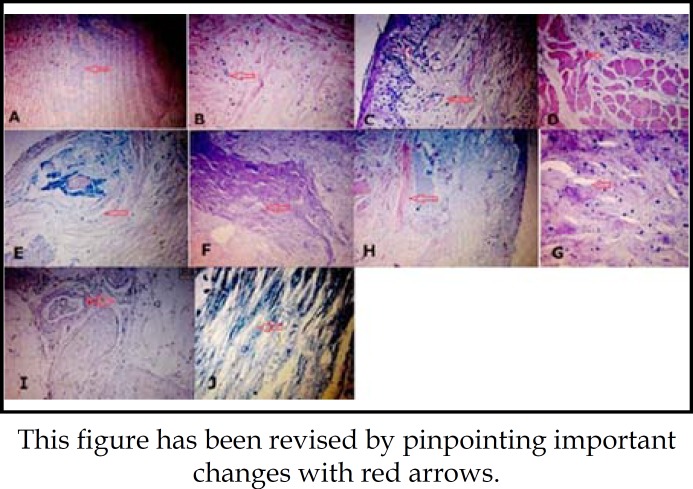
Electron microscope images of sections. (A) Specimen section №2 HE staining ×100. (B) Specimen section №3 HE staining ×100. (C) Specimen section №18 HE staining ×100. (D) Specimen section №46 HE staining ×100.(E) Specimen section №48 HE staining ×100. (F) Specimen section №35 HE staining ×25. (G) Specimen section №35 HE staining ×400. (H) Specimen section №55 HE staining ×25. (I) Specimen section №53 HE staining ×100. (J) Specimen section №53 Spielmeyer method staining ×400

**Table-I:** Immune peroxidase chemical reaction tests results

	*№* * 2*	*№* * 3*	*№* * 18*	*№* * 46*	*№* * 48*	*№* * 35*	*№* * 36*	*№* * 55*	*№* * 53*
LCA	-	+	+	-	+	+	+	++	-
CD-3 CD-20	+	+	+	+	+	+	+	++	+
CD-4	-	-	+	-	+	+	+	++	-
CD-8	-	-	+	-	+	+	+	++	-
CD-56	+	-	+	-	+	+	+	++	+

## RESULTS

The imaging characteristics under the electron microscope are shown in (Fig.2A). About 10% of the tissue sections had epithelial hyperplasia points with hyperkeratosis and acanthosis.The images of basal oral mucosa and submucosa were clear without a large area of atrophy. Although tiny necrotic points could be observed, there was no inflammatory reaction (Fig.2B).

About 90% of the sections had epithelial atrophy points with different degrees of damage, and meanwhile had moderate infiltration of lymphocytes-macrophages in the basal oral mucosa (Fig.2C). Muscle fiber atrophy could be observed in deep submucosa with the manifestation of injury in the "neurological regulatory system", which was more obvious especially in the "non-hyperplasia" of and "already hyperplasia" muscle fiber bundles (Fig.2D).

The oral mucosa in the tissue sections of patients who died of cardiovascular diseases with a history of alcohol abuse changed similarly to the oral mucosa of those who did not have a long history of alcoholism. Nevertheless, about half of the sections showed that extensive necrotic points were observed in different parts of oral mucosa, accompanied by a secondary infection, trace amount of inflammatory reaction, as well as extension along the outer periphery of focus. It is worth noting that the necrosis was structurally featured in stratification and pagination (Fig.2E).

About 15% of the sections were of more dense and homogeneous necrotic tissue with microbial colonization and a small amount of filamented neutrophils and macrophages (Fig. 2F and 2G).The necrotic focus of 5% of the sections was located above the epithelial tissue, which did not differ distinctively from other tissues (Fig.2H).

In 48% of the sections were found damaged small nerve bundles in deep oral mucosa, accompanied by necrosis in neuron axon and its myelin membrane (Fig. 2I and 2J). Notably, 85% of the sections were subjected to hyaline degeneration in the capillary arterial vascular wall.Moreover, the immune peroxidase chemical reaction tests results are summarized in Table-I.

## DISCUSSION

Preliminary discussion was conducted herein concerning the impact of alcohol on oral mucosa. Alcohol can destroy the lipid composition that the protective layer of oral mucosa covers acanthosis granules, and disrupt the normal order of epithelial lipid molecules, resulting in a gap between epithelial cells and increasing oral mucosal permeability. In other words, alcohol opens a pathway for deep soft tissues. Meanwhile, previous researches have confirmed that the most important risk factors in the development of oral cancer are smoking and alcohol.^3^Fiorettiet al^4^ found that alcoholic beverages manly accounted for the oral and pharyngeal cancers in non-smoking patients. There are several pathogenesis for ethanol acting on the oral mucosa. Active oxidation may directly undermined DNA. The invasive ability of carcinogen surrounding oral mucosa is increased, which relies on the enhanced solubility of the carcinogen or increased mucosal permeability.^5^ The carcinogenic effect of ethanol commonly occurs in case of daily ethanol intake higher than 45 ml.^6^

Acetaldehyde, the first metabolite of ethanol, has the largest carcinogenic potency. Acetaldehyde has been verified to be highly toxic, mutagenic and carcinogenic in different cell cultures and animal models.^7^Homann et al^7^ demonstrated increased salivary acetaldehyde levels even after ingesting moderate amounts of ethanol, allowing significant acetaldehyde accumulation in oral tissues during chronic ethanol consumption and may explain some of the cytologic anomalies in the oral mucosa of non-smoker alcoholics. Several researchers also reported some anomalies, including increased nuclear area,^8^ epithelial atrophy due to decreased basal cellular size,^7^ dysplastic changes with keratosis and increased number of mitotic figures.^9^ However, in the present study, the nucleus/cytoplasm ratio was not determined by the cytomorphometry. The exfoliative cytology of established oral cancers exhibited polyploid DNA profiles and reduced cytoplasmic area.^10^ Since polyploidy DNA profiles were discerned only in established oral cancer results, more subtle changes in DNA and nuclear morphologic characteristics may be observed in clinically normal mucosa, and may be just as crucial for detecting future cancer in susceptible patients as polyploidy is for detecting cancer.^11^

The presence of contaminants, such as polycyclic aromatic hydrocarbons and nitrosamines, is a significant carcinogenic factor in alcoholic beverages.^12^ Regardless, the total amount of ethanol and the duration of ethanol consumption may be more important than the type or composition of the alcoholic beverage consumed.^13^^,^^14^

All the above experimental studies have proven that the stratified structure of oral mucosa was damaged in the presence of alcohol, thus resulting in a gap between epithelial cells, increasing the permeability of oral mucosa, and facilitating deep penetration of carcinogen. Alcohol evidently promotes the development of oral cancer.

## CONCLUSION

According to the histopathological characteristics of the specimen sections obtained from 60 patients, we found that oral mucosa was altered under the stimulation of alcohol. A half of the specimen sections could be described as necrosis, especially the lesion in the basal oral mucosa was far more dramatic. The extent of necrosis, however, was not exactly the same. A small number of sections were subjected to only small necrotic points with a small amount of hyperkeratosis and acanthosis, and no inflammatory reaction was found. While the other half were of necrotic points that were rendered as "stratification". Muscle fibers underwent atrophy deep in the submucosa in part of specimen sections, as well as the necrosis of neuron axon and myelin membrane. In summary, the stratified structures of oral mucosa were susceptible to different degrees of injury in the presence of alcohol. The epithelial layer of oral mucosa was manifested as epithelial hyperplasia points with hyperkeratosis and acanthosis, as well as part of necrosis. Not only necrotic points were found in the basal oral mucosa and submucosa, but also atrophy muscle fiber atrophy was observed in deep submucosa with injury in the "neurological regulatory system".

## Authors Contributions:

FL: Designed the protocol and prepared the final manuscript.

WLL: Clinical data collection and experiments.
